# Synergistic Effects of a Pro-Inflammatory–High-Fat Composite Dietary Pattern on Gut–Liver Injury and the Therapeutic Potential of *Haematococcus pluvialis*-Derived Astaxanthin

**DOI:** 10.3390/nu18071048

**Published:** 2026-03-25

**Authors:** Jing Feng, Chao Han, Jinpeng Zhao, Zhuo Yang, Chen Chen, Rongzi Li, Chaoqun Sun, Liyuan Wang, Junsheng Huo, Shi Shen, Qin Zhuo

**Affiliations:** Key Laboratory of Public Nutrition and Health, National Institute for Nutrition and Health, Chinese Center for Disease Control and Prevention, Beijing 100050, China; fengjing0921@163.com (J.F.); hanchao@ninh.chinacdc.cn (C.H.); zhaojp@ninh.chinacdc.cn (J.Z.); yangzhuo@ninh.chinacdc.cn (Z.Y.); chenchen@ninh.chinacdc.cn (C.C.); lirz@ninh.chinacdc.cn (R.L.); suncq@ninh.chinacdc.cn (C.S.); wangly@ninh.chinacdc.cn (L.W.); huojs@ninh.chinacdc.cn (J.H.); shenshi@ninh.chinacdc.cn (S.S.)

**Keywords:** gut–liver axis, NHANES, dietary inflammatory index, high-fat diet, astaxanthin, organoids, enterohepatic comorbidity

## Abstract

**Background and Objectives:** Pro-inflammatory diet and high-fat diet (HFD) often coexist in real-world, but their combined impact on the gut–liver axis and potential nutritional countermeasures remain insufficiently studied. This study aimed to evaluate a pro-inflammatory–high-fat composite dietary pattern on the intestine and liver in the population, and to further evaluate the protective potential of astaxanthin (ATX) in complementary experimental systems. **Methods:** Data from the NHANES 2005–2010 were used to construct four composite exposure groups based on the dietary inflammation index (DII) and energy from fat. Survey-weighted regression analyses were performed to examine associations with systemic inflammation and liver injury. Interaction and *C*-reactive protein (CRP)-mediated effect analyses were conducted. Fifty SD rats were randomly divided into control group, model group induced by HFD combined with inflammatory factors, and low-, medium-, and high-dose *Haematococcus pluvialis* (HP) intervention groups. Serum lipids, liver enzymes, liver and colon pathology, and inflammatory and oxidative markers were measured in rats. In an in vitro organ-on-chip barrier model, the effect of ATX was observed when colonic barrier damage was induced using palmitic acid and lipopolysaccharides. **Results:** The high DII combined with HFD showed the largest increases in CRP, liver enzymes, and fatty liver index. A synergistic interaction was observed between DII and HFD, with CRP mediating approximately 20% of the effect. In rat model, HP-derived ATX improved the lipid profile, attenuated hepatic steatosis and oxidative damage, and reduced colonic pro-inflammatory cytokines, while restoration of tight junction proteins was limited. In colon organoid model, ATX showed limited efficacy in improving inflammation and barrier function. **Conclusions:** The pro-inflammatory–high-fat dietary pattern synergistically exacerbates gut–liver dysfunction. HP-derived ATX alleviates metabolic and inflammation-induced enterohepatic comorbidity, but its effect on repairing barrier structure is limited.

## 1. Introduction

In recent years, the incidence of intestinal and liver diseases has continued to rise worldwide, becoming a major public health challenge [1,2]. As core organs that maintain metabolic and immune homeostasis, the intestine and liver are tightly connected through the gut–liver axis. Chronic inflammation or metabolic dysregulation in either organ may amplify through this axis, culminating in enterohepatic comorbidity [3]. Diet is one of the most important and modifiable factors influencing the gut–liver axis. In particular, a pro-inflammatory diet is a key link between unhealthy dietary patterns and metabolic diseases [4]. To quantitatively assess the inflammatory potential of diet, the Dietary Inflammatory Index (DII) has been widely used and is significantly associated with systemic inflammation, metabolic disorders, and liver diseases [4,5,6]. Meanwhile, high-fat diet (HFD), a well-established health risk factor, can impair intestinal barrier integrity and disrupt hepatic metabolism by altering gut microbiota structure and inducing oxidative stress and inflammatory responses, thereby contributing to gut–liver comorbidity [7,8].

Current studies predominantly focused on either the inflammatory potential of diet or fat intake in relation to health outcomes alone. However, in real-world dietary contexts, pro-inflammatory and high-fat dietary patterns often co-occur. High-fat foods rich in saturated fat and sugars typically possess high pro-inflammatory potential, and these exposures may act synergistically to amplify adverse effects on the gut–liver axis [9]. Evidence on this “pro-inflammatory–high-fat” composite dietary pattern remains limited. In addition, current treatment of gut–liver comorbidity often targets a single organ or pathway, with limitations including heterogeneous efficacy, insufficient long-term safety, and poor adherence [10,11]. More importantly, many therapeutic strategies mainly target downstream inflammatory or metabolic manifestations, while inadequately correcting upstream events such as intestinal barrier damage, which may contribute to relapse after treatment discontinuation [9].

Astaxanthin (ATX) is a xanthophyll carotenoid with an amphiphilic structure, exhibiting potent antioxidant and anti-inflammatory activities [12,13]. *Haematococcus pluvialis* (HP) is an important natural source of ATX [14]. Studies suggest that ATX has protective potential in models of HFD associated fatty liver or chemically induced colitis [13,15]. However, under gut–liver comorbidity, the intervention characteristics and possible targets of HP-derived ATX still lack comprehensive verification. Intestinal related studies have predominantly focused on acute chemically induced colitis, whereas hepatic related studies commonly use single-etiology models such as high-fat or alcohol exposure. Few comorbidity models can truly recapitulate the clinically common coexistence of intestinal barrier injury and hepatic steatosis. Therefore, the preventive effects and potential targets of ATX in “pro-inflammatory–high-fat” diet induced enterohepatic comorbidity remain to be elucidated.

Therefore, this study integrated analyses of the National Health and Nutrition Examination Survey (NHANES), an HFD + DSS-induced rat model of concurrent intestinal and hepatic injury, and an in vitro colon organoid–immune cell barrier system. We aimed to elucidate the combined impact of a “pro-inflammatory–high-fat” dietary pattern on intestinal and hepatic health at the population level, and to evaluate the protective effects of HP-derived ATX in complementary experimental systems. By connecting population-level risk signals with experimental phenotypes and barrier-function, this study seeks to strengthen evidence for the diet–inflammation–metabolic disease causal chain and to provide a scientific basis for targeted dietary interventions for gut and liver health.

## 2. Materials and Methods

### 2.1. Study Design and Overall Framework

This study employed a multi-faceted approach. Firstly, data from the NHANES were analyzed to evaluate associations between the pro-inflammatory–high-fat dietary pattern and markers of systemic inflammation and liver injury. Secondly, a rat model of enterohepatic comorbidity was induced by combining HFD with a pro-inflammatory stimulus, and the effects of HP-derived ATX were evaluated. Finally, the direct impact of ATX on epithelial barrier function was investigated using an in vitro colon organoid–immune cell co-culture system.

### 2.2. NHANES Data and Indicator Definitions

Data were obtained from NHANES 2005 to 2010. The study population included adults aged 18–64 years. Individuals were excluded if they met any of the following criteria: (1) pregnancy; (2) missing data on exposure, outcomes, or key covariates; (3) implausible energy intake (males < 800 or >5000 kcal/day, females < 600 or >4000 kcal/day); (4) viral hepatitis, cirrhosis, or other diagnosed liver diseases; (5) excessive alcohol consumption (>210 g/week for males, >140 g/week for females).

**Figure 1:**
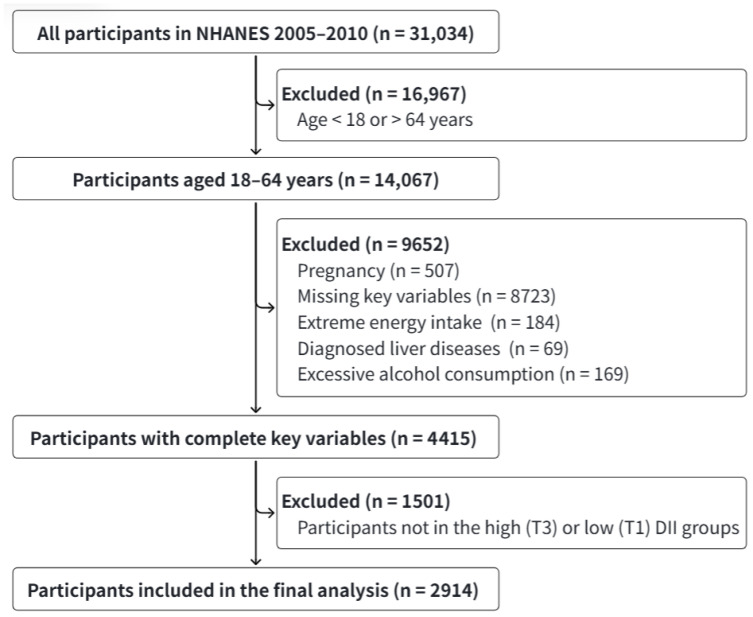
Flow chart for screening study participants.

Based on the protocol established by Shivappa et al. [5], the DII was calculated to quantify dietary inflammatory potential. The DII, based on 45 food parameters, scores an individual’s diet by comparing their intake of each component to a global reference database, reflecting its overall inflammatory impact. A higher (more positive) DII score indicates a more pro-inflammatory diet, typically rich in red meat, sugars, and saturated fats. A lower (more negative) score reflects a more anti-inflammatory diet, abundant in fruits, vegetables, and whole grains. The DII score ranges from −9 (highly anti-inflammatory) to +8 (highly pro-inflammatory), with a higher score indicating greater pro-inflammatory potential of the diet. DII scores were categorized by gender-specific tertiles (T1, T2, T3). To create a high-contrast exposure variable when combining DII with fat intake and to minimize group overlap, participants in the middle (T2) DII group were excluded from the composite dietary exposure grouping and analysis. Ultimately, 2914 eligible participants were included in the final analysis (Figure 1).

HFD was defined as energy from fat (%E) > 35%, consistent with the acceptable macronutrient distribution recommended by the Dietary Guidelines for Americans [16]. Participants were then classified into four mutually exclusive groups based on their DII tertile (T1/T3) and HFD status: Group 1: low DII (T1) and non-HFD; Group 2: high DII (T3) and non-HFD; Group 3: low DII (T1) and HFD; Group 4: high DII (T3) and HFD. Group 1 served as the reference group.

Systemic inflammation indicators included serum *C*-reactive protein (CRP) and white blood cell (WBC) count. History of gastrointestinal illness was obtained based on the NHANES household interview questionnaire (HSQ510-SP have stomach or intestinal illness?), with affirmative responses recorded as having had gastrointestinal illness. Liver injury indicators included serum alanine aminotransferase (ALT), aspartate aminotransferase (AST), and gamma-glutamyl transferase (GGT). Elevated liver enzymes were defined as ALT > 35 U/L for male and >25 U/L for female [17]. Hepatic steatosis was evaluated using the fatty liver index (FLI). According to Bedogni et al., hepatic steatosis was defined as FLI ≥ 60 [18]. Based on the criteria by Angulo et al. [19], the non-alcoholic fatty liver disease fibrosis score (NFS) ≥ −1.455 was used to identify participants at risk of progressive fibrosis requiring further assessment.

Covariates included demographic characteristics (age, sex, race, and education), lifestyle variables (smoking, physical activity, total energy intake, and alcohol intake), and metabolic factors (body mass index, waist circumference, diabetes, and hypertension status).

### 2.3. Animals and Experimental Design

Fifty specific pathogen-free male SD rats (6–8 weeks old; 350 ± 20 g) were purchased from Sibefu (Beijing, China) and housed in the Nanhwei Road Animal Laboratory of the Chinese Center for Disease Control and Prevention. The housing conditions included a temperature of 20–24 °C, relative humidity of 40–70%, a 12 h light/dark cycle, and 15 air changes per hour.

Rats were randomly assigned to five groups based on body weight (BW) (*n* = 10/group): control (CON), model (MOD), and low-, medium-, and high-dose HP powder groups (LHP, MHP, and HHP). HP powder (2.32% ATX; Alphy, Chuxiong, China) was administered at doses of 66.7, 133.3, and 400.0 mg/kg BW for LHP, MHP, and HHP groups, respectively. These doses were determined with reference to the recommended intake of HP from the National Health Commission of China (≤ 0.8 g/day) and the requirements of the Technical Guidelines for Functional Testing and Evaluation of Health Foods (2023) [20,21]. Based on our experience and previous studies [22], an 8-week intervention with a HFD (45% energy from fat) combined with 1.5% DSS in drinking water induced hepato-intestinal injury in rats. Rats in the CON group were fed a standard diet (D12450H; 10% energy from fat); rats in the MOD group were fed a HFD (D12451; 45% energy from fat). Rats in the LHP, MHP, and HHP groups were fed the HFD supplemented with 0.08%, 0.16%, and 0.48% HP, respectively. All diets were purchased from Xiaoshuyoutai (Beijing, China). Rats in the CON group received sterile water, whereas the other four groups received 1.5% dextran sulfate sodium (DSS; 36–50 kDa, Meilun, Dalian, China) in drinking water for 1 week followed by sterile water for 1 week, for 4 cycles (8 weeks total).

**Figure 2:**
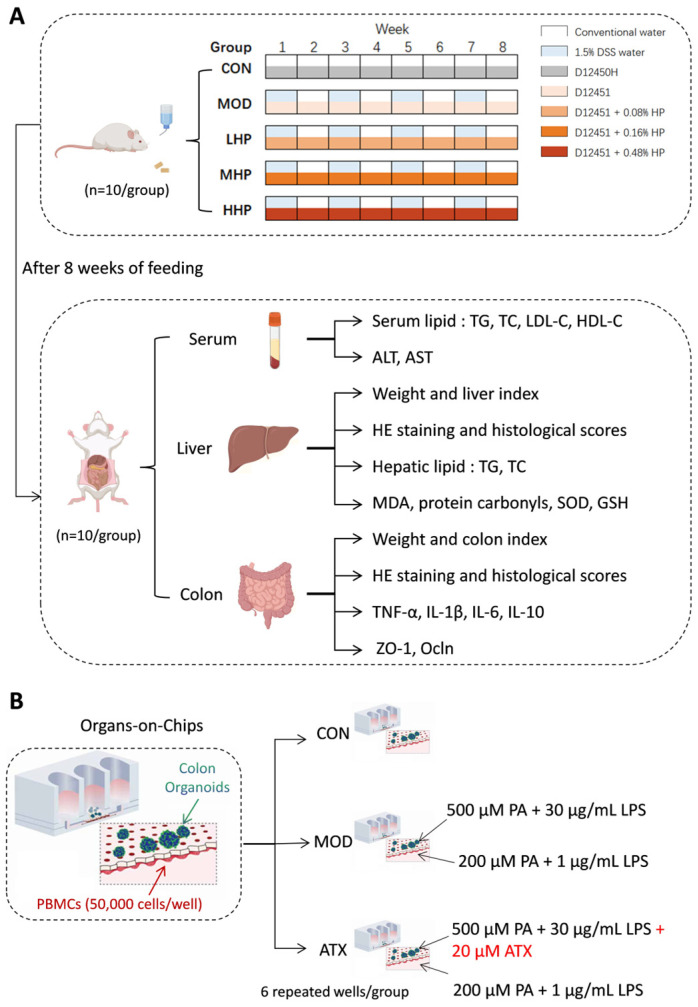
Procedures for experiments in vitro and in vivo. (**A**) Experimental design in rats. (**B**) Experimental design in colon organoids–immune cell co-culture. PA, palmitic acid; LPS, lipopolysaccharide; ATX, astaxanthin.

Rats were housed two per cage with ad libitum access to food and water. Body weight and food intake were recorded weekly. After the 8-week intervention, rats were fasted for 12 h, anesthetized, and blood was collected for serum separation. Liver and colon tissues were harvested for subsequent analysis (Figure 2A).

Serum was analyzed for triglycerides (TG; Sjodax, Beijing, China), total cholesterol (TC; Sjodax, Beijing, China), low-density lipoprotein cholesterol (LDL-C; Sjodax, Beijing, China), high-density lipoprotein cholesterol (HDL-C; Sjodax, Beijing, China), ALT (Sjodax, Beijing, China), and AST (Sjodax, Beijing, China). Hepatic homogenates were assessed for TG and TC, malondialdehyde (MDA; Jiancheng, Nanjing, China), protein carbonyls (Jiancheng, Nanjing, China), superoxide dismutase (SOD; Sjodax, Beijing, China), and reduced glutathione (GSH; Solarbio, Beijing, China). In colon tissue, levels of tumor necrosis factor-α (TNF-α; Mlbio, Shanghai, China), interleukin-1β (IL-1β; Mlbio, Shanghai, China), interleukin-6 (IL-6; Mlbio, Shanghai, China), interleukin-10 (IL-10; Mlbio, Shanghai, China), and the tight junction proteins zonula occludens-1 (ZO-1; CUSABIO, Houston, TX, USA) and occludin (Ocln; CUSABIO, Houston, TX, USA) were quantified by ELISA. Hematoxylin-eosin (HE) staining was performed on the liver and colon, and histological scoring was performed by a pathologist in a blinded manner.

All procedures of this experiment have been approved by the Ethics Committee of the Institute of Nutrition and Health, Chinese Center for Disease Control and Prevention (No. 2024-NINH-IACUC-041).

### 2.4. Colon Organoids–Immune Cell Co-Culture

A colon organoid–immune cell co-culture barrier model was cultured on an organ-on-chip platform (IBAC M1; Daxiang, Beijing, China). Colon organoids were seeded onto a porous membrane to form a continuous epithelial layer. Peripheral blood mononuclear cells (PBMCs; Milecell, Shanghai, China) were then seeded on the basolateral side (50,000 cells/well) to mimic epithelial–immune interactions in a mucosal barrier microenvironment. Six repeated wells were used for each group (Figure 2B).

Three experimental groups were established: (1) a control (CON) group with culture medium only; (2) a model (MOD) group stimulated with palmitic acid (PA; Yuanye, Shanghai, China) and lipopolysaccharide (LPS; MCE, Monmouth Junction, NJ, USA), applied at 500 μM PA + 30 μg/mL LPS on the apical side and 200 μM PA + 1 μg/mL LPS on the basolateral side; (3) an ATX group receiving the same PA/LPS stimulation together with 20 μM ATX (MCE, Monmouth Junction, NJ, USA). The concentrations and stimulation duration were selected based on preliminary optimization experiments, and the detailed stepwise culture procedures are provided in the Supplementary Materials.

Barrier function was assessed by transepithelial electrical resistance (TEER) and Yellow P tracer assay. Decreased TEER values or increased Yellow P flux indicated impaired barrier function. Bright-field images were acquired on days 3 to 5. At day 5, RNA was collected and the expression levels of ZO-1, Ocln, TNF-α, IL-1β, and IL-6 in colonic organoids were quantified by RT–qPCR. Additional technical details are described in the Supplementary Materials.

### 2.5. Statistical Analysis

All analyses were performed using RStudio (version 4.5.0; Posit PBC, Boston, MA, USA) and Stata (version 17.0; StataCorp LLC, College Station, TX, USA). GraphPad Prism (version 8.0; GraphPad Software, Inc., San Diego, CA, USA) was used for plotting. Statistical significance was defined as *p* < 0.05.

NHANES analyses incorporated appropriate survey weights to account for the complex sampling design. Weighted linear regression was used for continuous outcomes and weighted logistic regression for binary outcomes, with stepwise adjustment for potential confounders. The synergistic effect between the DII and high-fat intake was examined through interaction term testing. Exploratory mediation analyses were conducted to assess whether CRP, as a single biomarker-based indicator of inflammatory, statistically mediated the association between the combined exposure and indicators of liver injury.

To test robustness and address potential confounding or reverse causality, we conducted four types of sensitivity analyses: (1) recalculating DII using the second-day dietary recall data; (2) including previously excluded participants with liver disease; (3) repeating analyses using energy-adjusted DII scores; and (4) redefining the composite exposure as a continuous term of DII and %E.

For experimental data, normality and homogeneity were assessed. Variables meeting both assumptions were analyzed using one-way ANOVA with Tukey’s HSD post hoc tests, whereas variables violating these assumptions were analyzed using Kruskal–Wallis tests with Dunn’s multiple comparisons.

## 3. Results

### 3.1. Baseline Characteristics of Participants

Table 1 summarizes the baseline characteristics of the 2914 participants according to the composite dietary pattern groups. Significant intergroup differences were observed in demographic, anthropometric, and dietary indicators (*p* < 0.05) but not in baseline levels of inflammatory biomarkers, liver enzymes, and liver health indices (*p* > 0.05).

Demographically, female and middle-aged adults were more prevalent in the HFD groups (Groups 3 and 4), whereas male and younger adults were more common in the non-HFD groups (Groups 1 and 2). Regarding anthropometry, Group 3 had the highest BMI and waist circumference, significantly exceeding the values in the non-HFD groups. As expected, total fat intake and the %E (both > 41%) were markedly higher in the HFD groups than in the non-HFD groups (approximately 27%).

The absence of significant differences in CRP, WBC, ALT, AST, GGT, and FLI at baseline minimizes potential confounding for the subsequent analyses of dietary effects (all *p* > 0.05; Table 1).

### 3.2. Linear Regression of Composite Exposure Groups and Outcomes

Across three progressively adjusted models (Table 2), positive associations were observed between all composite exposure groups and the outcomes. In Model 1, Group 4 showed the strongest associations with all outcomes, followed by Group 3, while Group 2 showed the weakest yet still significant associations (all *p* < 0.05). After further adjustment for lifestyle factors in Model 2, the β coefficients decreased by approximately 7–10% relative to Model 1, while all associations remained significant (all *p* < 0.05). In Further adjustment for metabolic factors in Model 3 resulted in an additional attenuation, with β coefficients for most core indicators decreasing by 12–16% relative to Model 1 (all *p* < 0.05). Overall, the pro-inflammatory–high-fat dietary pattern demonstrated the strongest association with inflammatory markers and adverse liver health indicators.

### 3.3. Logistic Regressions of Composite Exposure Groups and Outcomes

After adjustment for demographic, lifestyle, and metabolic factors, weighted logistic regression analysis showed that the composite exposure groups were positively associated with elevated liver enzymes and with significant steatosis or fibrosis (Table 3). Although the ORs showed modest attenuation with stepwise adjustment, a consistent gradient in association strength was observed. Group 4 had the strongest association, followed by Group 3, and then Group 2 (all *p* < 0.05).

Furthermore, interaction analysis further indicated a positive multiplicative interaction between DII and HFD, suggesting a synergistic adverse effects on liver health (Table 4). This association remained robust and independent of confounders.

### 3.4. Mediating Role of CRP Between the Groups and ALT/FLI

**Figure 3:**
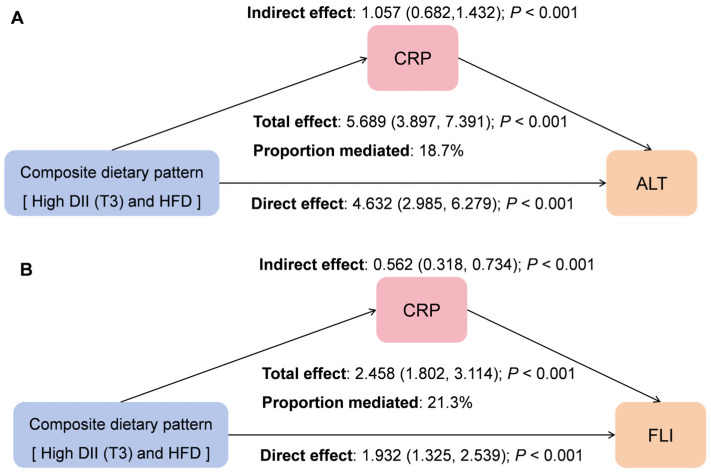
Mediation patterns between composite dietary patterns (Group 4 vs. Group 1) and CRP, ALT, or FLI. (**A**) Composite dietary patterns and ALT. (**B**) Composite dietary patterns and FLI. Note: CRP, *C*-reactive protein; ALT, alanine aminotransferase; FLI, Fatty liver index. The arrow s indicate direct or indirect effects.

To explore the potential role of systemic inflammation, we performed a mediation analysis using serum CRP as the mediator. In fully adjusted model, CRP demonstrated a significant mediating effect on the associations between the pro-inflammatory–high-fat diet (Group 4 vs. Group 1) and both elevated ALT and higher FLI The *p* values for the total, indirect, and direct effects were all <0.05 (Figure 3).

### 3.5. Sensitivity Analyses of Population Data

As shown in Table 5, all sensitivity analyses were consistent with the primary analysis, showing significant positive associations (all *p* < 0.05). In the first three analyses, ORs for associations with CRP, ALT, and FLI were largely consistent with the primary estimates. In the fourth analysis of continuous terms, significantly positive β coefficients were observed for all three outcomes. These results indicated that the associations of the pro-inflammatory–high-fat composite dietary pattern with CRP, ALT, and FLI were robust. The associations were not affected by dietary assessment methods, potential reverse causality, energy intake levels, or exposure variable definitions.

### 3.6. Body Weight and Food Intake of Rats

**Figure 4:**
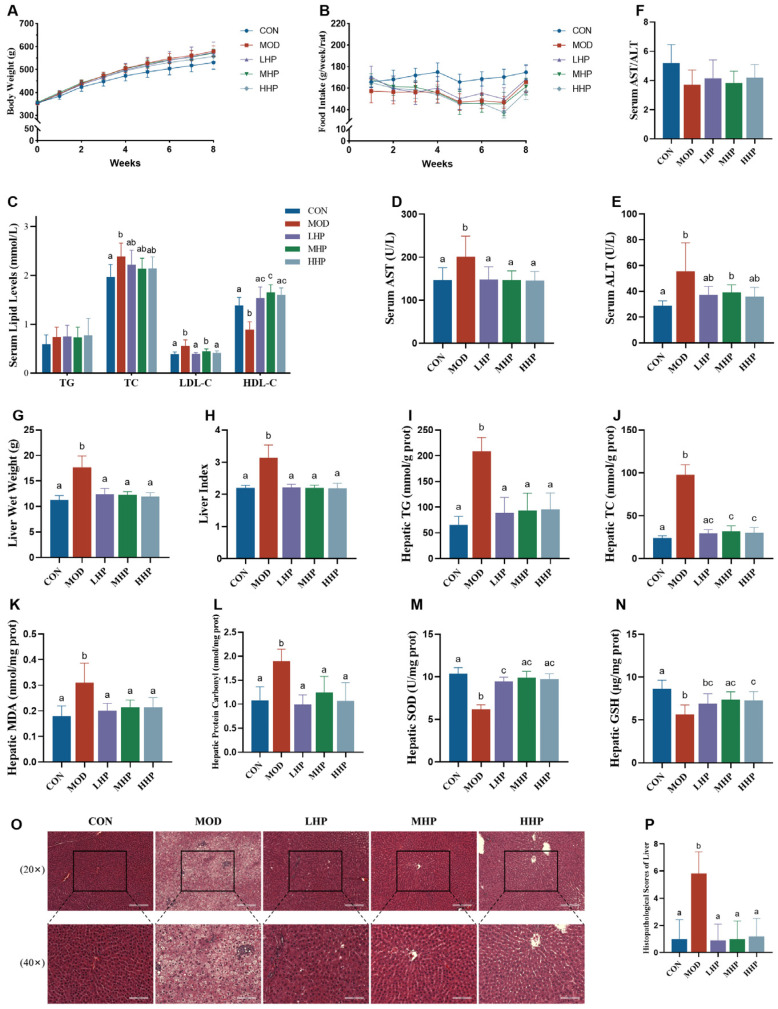
Evaluation of body weight, food intake, serum lipid levels and liver health status of rats in each group. (**A**) Weekly body weight; (**B**) weekly food intake; (**C**) serum lipid levels; (**D**) serum AST level; (**E**) serum ALT level; (**F**) serum AST/ALT ratio; (**G**) liver wet weight; (**H**) liver index; (**I**) hepatic TG level; (**J**) hepatic TC level; (**K**) hepatic MDA level; (**L**) hepatic protein carbonyl level; (**M**) hepatic SOD level; (**N**) hepatic GSH level; (**O**) HE staining of liver; (**P**) histopathological scores of liver. Note: TG, triglycerides; TC, total cholesterol; LDL-C, low-density lipoprotein cholesterol; HDL-C, high-density lipoprotein cholesterol; AST, aspartate aminotransferase; ALT, alanine transaminase; MDA, malondialdehyde; SOD, superoxide dismutase; GSH, reduced glutathione. Different letters indicate statistically significant differences among groups (*p* < 0.05).

Compared with the CON group, rats fed HFD had lower weekly and total food intake as well as a lower food efficiency ratio, but a higher total energy intake (*p* < 0.05; Figure 4A,B, Table 6). Body weights from week 3 onward and total weight gain were higher in the MOD, LHP, and MHP groups than in the CON group (*p* < 0.05). No significant differences were observed among the intervention groups and the MOD group (*p* > 0.05).

### 3.7. Effects of HP-Derived ATX on Serum Biochemical Indices in Rats

As shown in Figure 4C and Supplementary Table S1, serum TC and LDL-C levels were higher and HDL-C levels were lower in MOD than in CON (all *p* < 0.05). HP supplementation at different doses reduced LDL-C and increased HDL-C levels to varying degrees (*p* < 0.05). No significant improvement in TG levels was observed (*p* > 0.05).

Regarding liver enzymes (Figure 4D–F), serum ALT and AST levels were significantly increased in the MOD group relative to the CON group. Compared with the MOD group, AST levels were decreased by HP interventions at all doses (*p* < 0.05). In contrast, no significant change in ALT levels was observed (*p* > 0.05).

### 3.8. Effects of HP-Derived ATX on Hepatic Lipids Accumulation and Oxidative Stress in Rats

As shown in Figure 4G,H and Supplementary Table S2, liver wet weight and liver index were significantly increased in the MOD group compared with CON group, and ATX intervention at all doses reduced these parameters to levels comparable to the CON group (*p* < 0.05).

Additionally, as shown in Figure 4I–N, rats in the MOD group also showed increased hepatic lipid levels (TG, TC), lipid peroxidation products (MDA), and protein oxidation products (protein carbonyls), with decreased antioxidant enzyme (SOD) and non-enzymatic antioxidant (GSH) levels (*p* < 0.05). Compared with MOD, all three intervention groups significantly reduced hepatic TG, TC, MDA, and protein carbonyl levels and increased SOD levels (*p* < 0.05), with no significant differences among interventions (*p* > 0.05). The MHP and HHP groups also increased GSH levels (*p* < 0.05).

Histological assessment supported these findings. HE staining indicated marked hepatic steatosis and mild inflammation in the MOD, accompanied by significantly increased histological scores; all HP intervention groups attenuated hepatic injury and reduced histological scores (*p* < 0.05; Figure 4O,P).

### 3.9. Effects of HP-Derived ATX on Colonic Inflammation and Intestinal Barrier Integrity in Rats

**Figure 5:**
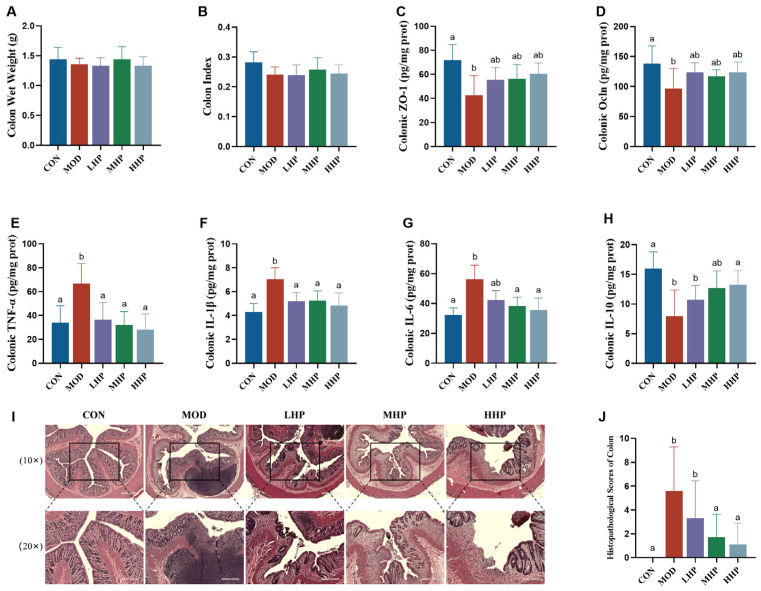
Colon health status of rats in each group. (**A**) Colon wet weight; (**B**) colon index; (**C**) colonic levels; (**D**) colonic levels; (**E**) colonic levels; (**F**) colonic levels; (**G**) colonic levels; (**H**) colonic levels; (**I**) HE stain-ing of colon; (**J**) histopathological scores of colon. Note: ZO-1, zonula occludens-1; Ocln, occludin; TNF-α, tumor necrosis factor alpha; IL-1β, inter-leukin 1 beta; IL-6, interleukin 6; IL-10, interleukin 10. Different letters in each row indicate statis-tically significant differences among groups (*p* < 0.05).

As shown in Figure 5A,B and Supplementary Table S3, no significant differences were observed among groups in colon wet weight or colon index (*p* > 0.05).

Colonic levels of ZO-1 and Ocln were decreased in the MOD group compared to the CON group, while the three intervention groups did not differ significantly from the MOD group (*p* > 0.05; Figure 5C,D). The MOD group also had significantly higher levels of the pro-inflammatory cytokines TNF-α, IL-1β, and IL-6, and a lower level of the anti-inflammatory cytokine IL-10, compared to the CON group (all *p* < 0.05). All three intervention groups effectively normalized the levels of TNF-α and IL-1β. Additionally, the MHP and HHP groups also reduced IL-6 and increased IL-10 levels (*p* < 0.05; Figure 5E–H).

HE staining (Figure 5I) revealed pronounced inflammatory infiltration, crypt damage, edema, and fibrous connective tissue hyperplasia in colon tissues of MOD rats, resulting in significantly increased histological scores (*p* < 0.05). In the LHP group, crypt damage, edema, and fibrosis remained evident, and occasional individuals exhibited mild inflammatory infiltration; in the MHP and HHP groups, no significant inflammatory infiltration was observed, but fibrotic injury persisted. Consequently, histological scores were decreased in the MHP and HHP groups compared to the MOD group (*p* < 0.05; Figure 5J).

### 3.10. Inflammation and Barrier Integrity in Colon Organoids

**Figure 6:**
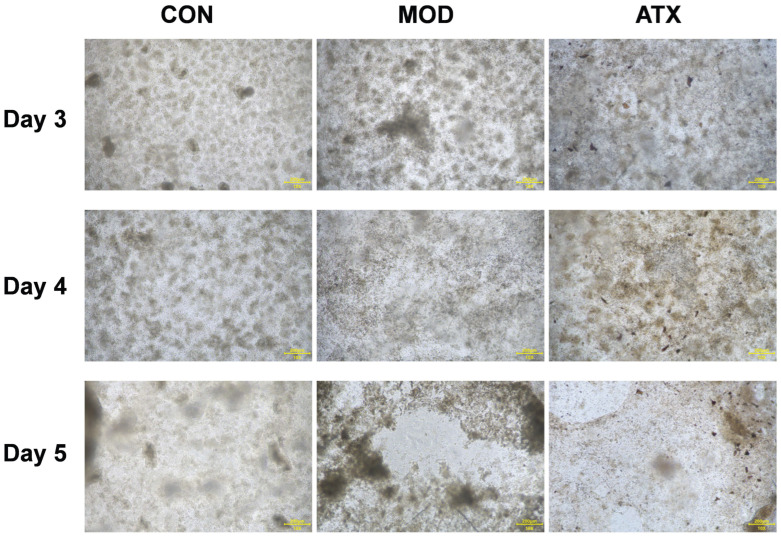
Typical illustration of light fields observed in colon organoids on days 3–5.

LPS/PA stimulation significantly compromised epithelial barrier function, as demonstrated by a marked decrease in TEER and a concurrent increase in Yellow P permeability in the MOD group compared to the CON group (*p* < 0.05; Table 7 and Figure 6). Compared with MOD, the ATX treatment significantly attenuated Yellow P (*p* < 0.05), whereas no significant difference was observed in TEER (*p* > 0.05).

The results of RT-qPCR showed that the relative levels of TNF-α and IL-1β in the colonic barrier were significantly increased after LPS/PA stimulation (*p* < 0.05). ATX treatment showed a trend toward lowering TNF-α, but the difference was not statistically significant (*p* > 0.05).

## 4. Discussion

This study innovatively applied a composite dietary exposure model combining DII and HFD in a population-based sample. The pro-inflammatory–high-fat dietary pattern (high DII and HFD) was associated with gut–liver injury, with the two factors exhibiting synergistic adverse effects. Moreover, this association was independent of key demographic, lifestyle, and metabolic confounders. The adverse synergy observed at the population level was mechanistically recapitulated in a rat model of comorbidity. Simultaneous exposure to high-fat and pro-inflammatory stimuli induced a comorbid phenotype characterized by synchronous colonic inflammation, barrier disruption, hepatic steatosis, and oxidative damage. HP-derived ATX was found to ameliorate this comorbid injury to some extent. Consistently, an in vitro colon organoid–immune cell co-culture system showed that combined metabolic–inflammatory stress can directly impair epithelial barrier function.

Exploratory mediation analysis suggested that CRP statistically accounted for part of the associations between the composite dietary exposure and liver injury (ALT/FLI), indicating a potential “pro-inflammatory–high-fat diet → systemic inflammation → liver injury” pathway. While previous studies have confirmed a significant positive correlation between the DII and CRP levels, the mediating role of CRP between diet and liver health was not explored [6]. Concurrently, significant changes in oxidative stress indices in the rat model may explain residual effects not mediated by CRP. These findings align closely with the gut–liver axis framework. Inflammatory challenge or HFD may alter microbial metabolism, weaken barrier integrity, and increase endotoxin translocation, thereby activating innate immune and inflammatory pathways and accelerating hepatic lipid deposition and inflammation [11,23,24].

Oxidative stress and lipid peroxidation are key drivers in the progression from hepatic steatosis to inflammation and fibrosis [25,26]. In our rat model of intestinal and hepatic co-injury, HP-derived ATX alleviated hepatic lipid deposition and oxidative damage independently of weight change, suggesting its benefits were more likely mediated through redox homeostasis and inflammatory tone rather than weight reduction. This is supported by evidence that medium- to high-dose ATX can regulate metabolic disorders without consistently affecting body weight [27]. ATX is thus more likely to reduce the risk of organ damage by improving lipid transport, redox homeostasis, and inflammatory responses [28]. Furthermore, the lack of effect on TC is notable. Recent studies have also shown that ATX can improve HDL-C levels, although its effects on other lipid parameters are heterogeneous [27,29].

A similar pattern was observed in the intestines. HP-derived ATX reduced the mucosal inflammatory microenvironment in rats. Studies also observed that ATX alleviated colonic inflammation and tissue damage in a DSS-induced mouse colitis model, which is consistent with the present findings [13,30]. However, restoration of tight junction protein expression was limited. This divergence between inflammatory improvement and incomplete structural recovery is biologically plausible, as full epithelial repair requires not only cytokine suppression but also matrix remodeling and cellular differentiation [31]. The combined exposure of HFD and DSS may induce more persistent fibrosis and tissue remodeling, making it difficult to yield detectable differences in tight-junction protein expression in the short-term interventions.

In the colon organoid model, ATX only partially reduced permeability (Yellow P), with limited effects on TEER and inflammatory factors. Several factors may explain this discrepancy with the in vivo findings. Firstly, whole HP powder contains additional bioactive constituents beyond ATX that may contribute to its in vivo efficacy. Secondly, the in vitro system lacks key elements present in vivo, such as gut microbiota, gut–liver axis signaling, and dynamic immune cell interactions. Recent studies have proposed that the synergy between ATX and the microbiota can enhance intestinal barrier defense through metabolites (e.g., short-chain fatty acids, bile acids) and signaling pathways such as PPARγ [30]. Thirdly, the lipophilic nature of ATX may limit its dispersion and effective exposure to epithelial cells in standard in vitro cultures. Several researchers have improved the bioavailability and developed colon-targeted nanodelivery systems to increase the effective exposure dose of ATX, thereby promoting the recovery of tight junction proteins [13,32]. Overall, the full effects of ATX appear closely linked to the gut microbiome, liver-derived factors, and target-site exposure.

The daily ATX intake in our rat study was estimated at approximately 0.87, 1.69, and 5.07 mg/kg BW in the low-, medium-, and high-dose groups, respectively. Converted based on the equivalent body-surface area index, human equivalent doses were estimated at approximately 8.46, 16.44, and 49.33 mg/d (for 60 kg adult) [33]. The dose design encompasses the recommended daily ATX intake for adults (12 mg/d/60 kg) [34]. Our high dose (5.07 mg/kg) is comparable to the 6 mg/kg dose shown to improve hepatic lipid deposition and oxidative stress–related indicators in HFD-fed rats [35]. Currently, few studies have evaluated the effects of ATX on intestinal barrier function in rats. Yilmaz et al. reported that ATX at 10 mg/kg BW could reduce intestinal damage induced by ischemia-reperfusion [36]. In a DSS-induced ulcerative colitis mouse model, Wei et al. found that ATX at 100 mg/kg BW significantly alleviated intestinal inflammation and barrier damage, and the observed effects were independent of the gut microbiota [37]. Zhang et al. reduced the effective dose to 1 mg/kg BW by ATX-loaded nanoparticles and still observed ameliorative effects in DSS-induced colitis in mouse [38]. Overall, compared with previous studies in rats, the doses used in this study fall within a relatively low interval. The low and medium doses in this study were closer to the feasible conversion range of common supplemental doses, while the high dose was close to the effective doses reported in some previous studies.

By integrating NHANES data, an in vivo comorbidity model, and an in vitro barrier system, this study builds a multi-level evidence chain linking high-fat and pro-inflammatory stimuli to experimental phenotypes. This design partially mitigates the temporal limitations inherent in cross-sectional analyses and provides a testable framework for mechanisms along the gut–liver axis under composite dietary exposure. This study has several limitations. Firstly, the NHANES analysis is cross-sectional and based on 24-h dietary recall, limiting causal inference and complete capture of dietary patterns. Secondly, CRP was measured at a single time point and therefore reflects only a partial biomarker-based indicator of inflammatory involvement. Thirdly, while the HFD + DSS rat model usefully recapitulates concurrent gut–liver injury, DSS is a stronger and more acute trigger than real-world dietary inflammation and therefore limits direct translational comparability with the human exposure pattern. Fourthly, the animal experiments did not include direct measurements of microbiota composition, bile acid metabolism, and related gut–liver mediators. Finally, ATX delivery and exposure comparability in the organoid system require further optimization. Despite these limitations, the convergence of evidence from human, animal, and in vitro models provides a useful multi-level framework for understanding how composite dietary exposures may influence gut–liver health and highlights ATX as a promising nutraceutical with protective potential.

## 5. Conclusions

This study supports that a pro-inflammatory–high-fat composite dietary pattern is associated with increased systemic inflammation and liver injury in adults, with evidence of synergistic adverse effect between DII and HFD. Complementary experimental models showed that supplementation with HP-derived ATX ameliorated metabolic disturbances and gut–liver comorbidity in an HFD + DSS-induced rat model, although its effects on restoring structural barrier proteins were limited. Therefore, maintaining gut–liver axis health may require simultaneous reduction in dietary inflammatory potential and energy from fat, and ATX may represent a promising functional factor for modulating gut–liver comorbidity.

## Figures and Tables

**Table 1:** Basic characteristics of the study population.

Variables	Category	Total (*n* = 2914)	Group 1 (*n* = 807)	Group 2 (*n* = 846)	Group 3 (*n* = 655)	Group 4 (*n* = 606)	*p*
Sex	Male	1513 (51.5%)	425 (50.0%)	456 (53.2%)	355 (56.5%)	277 (45.9%)	<0.001
	Female	1401 (48.5%)	382 (50.0%)	390 (46.8%)	300 (43.5%)	329 (54.1%)	
Age	Young adults	1627 (53.7%)	487 (58.7%)	479 (57.8%)	347 (49.3%)	314 (46.9%)	
	Middle-aged adults	1282 (46.3%)	318 (41.3%)	365 (42.2%)	307 (50.7%)	292 (53.1%)	
Race	Non-Hispanic White	1221 (67.8%)	316 (65.8%)	318 (62.6%)	301 (71.8%)	286 (72.3%)	<0.001
	Mexican American	647 (9.6%)	220 (12.4%)	197 (10.5%)	117 (7.0%)	113 (7.9%)	
	Non-Hispanic Black	634 (11.6%)	150 (10.2%)	182 (12.4%)	163 (12.5%)	139 (11.3%)	
	Other Hispanic	282 (5.1%)	82 (5.3%)	110 (7.9%)	47 (3.5%)	43 (3.2%)	
	Other race	130 (5.9%)	39 (6.4%)	39 (6.6%)	27 (5.2%)	25 (5.2%)	
Educational level	Less than high school	1565 (42.9%)	446 (43.9%)	478 (45.6%)	343 (42.7%)	298 (38.7%)	<0.001
	High school	765 (28.3%)	203 (26.6%)	194 (24.2%)	195 (32.6%)	173 (30.7%)	
	College or above	584 (28.8%)	158 (29.4%)	174 (30.2%)	117 (24.8%)	135 (30.6%)	
Gastrointestinal disease	No	2648 (90.7%)	735 (91.3%)	770 (90.5%)	585 (89.3%)	558 (91.8%)	<0.001
	Yes	266 (9.3%)	72 (8.7%)	76 (9.5%)	70 (10.7%)	48 (8.2%)	
Hypertension	No	2141 (73.7%)	612 (75.4%)	619 (73.4%)	469 (72.5%)	441 (73.1%)	<0.001
	Yes	773 (26.3%)	195 (24.6%)	227 (26.6%)	186 (27.5%)	165 (26.9%)	
Diabetes	No	2661 (93.4%)	753 (95.5%)	766 (92.3%)	594 (93.3%)	548 (92.4%)	<0.001
	Yes	253 (6.6%)	54 (4.5%)	80 (7.7%)	61 (6.7%)	58 (7.6%)	
Alcohol use	Yes	1714 (65.8%)	458 (64.5%)	485 (63.2%)	396 (66.6%)	375 (69.6%)	<0.001
	No	1200 (34.2%)	349 (35.5%)	361 (36.8%)	259 (33.4%)	231 (30.4%)	
Smoking status	Former smoker	1476 (52.4%)	430 (54.8%)	449 (54.9%)	291 (45.5%)	306 (53.4%)	<0.001
	Current smoker	1197 (43.9%)	313 (41.6%)	328 (40.7%)	301 (50.6%)	255 (43.6%)	
	Never smoker	241 (3.8%)	64 (3.6%)	69 (4.4%)	63 (3.9%)	45 (3.0%)	
Physical activity	Active	2047 (67.4%)	568 (67.0%)	620 (68.6%)	448 (66.9%)	411 (66.9%)	<0.001
	Sedentary	867 (32.6%)	239 (33.0%)	226 (31.4%)	207 (33.1%)	195 (33.1%)	
Alcohol intake (g/d)		3.32 ± 0.13	3.41 ± 0.25	3.56 ± 0.25	3.04 ± 0.25	3.21 ± 0.27	0.224
BMI (kg/m^2^)		28.54 ± 0.14	28.12 ± 0.27	28.18 ± 0.26	29.13 ± 0.3	28.87 ± 0.31	0.001
WBC (10^9^/L)		6.75 ± 0.04	6.75 ± 0.08	6.79 ± 0.08	6.83 ± 0.09	6.6 ± 0.09	0.208
ALT (U/L)		25.42 ± 0.29	24.93 ± 0.57	25.53 ± 0.52	25.59 ± 0.66	25.36 ± 0.58	0.852
AST (U/L)		24.46 ± 0.17	24.31 ± 0.33	24.65 ± 0.33	24.54 ± 0.35	24.21 ± 0.33	0.961
GGT (U/L)		25.41 ± 0.42	25.13 ± 0.8	26.03 ± 0.85	25.37 ± 0.79	24.44 ± 0.82	0.681
CRP (mg/L)		0.34 ± 0.01	0.32 ± 0.02	0.33 ± 0.02	0.36 ± 0.02	0.35 ± 0.02	0.461
Total energy intake (kcal/d)		2203.26 ± 18.9	2121.67 ± 35.81	2100.13 ± 35.78	2370.54 ± 39.44	2255.81 ± 38.96	<0.001
Total fat intake (g/d)		83.65 ± 0.89	65.89 ± 1.29	65.49 ± 1.3	109.66 ± 1.92	103.27 ± 1.84	<0.001
FLI		5.166 ± 0.049	5.012 ± 0.09	4.989 ± 0.091	5.397 ± 0.105	5.368 ± 0.108	<0.001

Note: Group 1: low DII (T1) and non-HFD; Group 2: high DII (T3) and non-HFD; Group 3: low DII (T1) and HFD; Group 4: high DII (T3) and HFD. WBC: White blood cell. ALT: Alanine aminotransferase. AST: Aspartate aminotransferase. GGT: Gamma-glutamyl transferase. CRP: *C*-reactive protein. FLI: Fatty liver index.

**Table 2:** Weighted linear regression analyses across composite exposure groups.

Variables	Groups	Model 1 β (95% CI)	*p*	Model 2 β (95% CI)	*p*	Model 3 β (95% CI)	*p*
WBC	Group 2	0.125 (0.032, 0.218)	0.008	0.118 (0.025, 0.211)	0.012	0.098 (0.012, 0.184)	0.025
	Group 3	0.213 (0.105, 0.321)	<0.001	0.198 (0.092, 0.304)	<0.001	0.176 (0.078, 0.274)	0.001
	Group 4	0.356 (0.248, 0.464)	<0.001	0.324 (0.218, 0.430)	<0.001	0.289 (0.185, 0.393)	<0.001
CRP	Group 2	0.452 (0.215, 0.689)	0.001	0.418 (0.189, 0.647)	0.001	0.385 (0.162, 0.608)	0.001
	Group 3	0.587 (0.352, 0.822)	<0.001	0.543 (0.318, 0.768)	<0.001	0.498 (0.285, 0.711)	<0.001
	Group 4	0.892 (0.658, 1.126)	<0.001	0.825 (0.602, 1.048)	<0.001	0.764 (0.558, 0.970)	<0.001
ALT	Group 2	2.158 (0.892, 3.424)	0.001	1.987 (0.785, 3.189)	0.001	1.765 (0.658, 2.872)	0.002
	Group 3	3.892 (2.568, 5.216)	<0.001	3.564 (2.358, 4.770)	<0.001	3.125 (2.048, 4.202)	<0.001
	Group 4	6.785 (4.892, 8.678)	<0.001	6.258 (4.485, 8.031)	<0.001	5.689 (3.987, 7.391)	<0.001
AST	Group 2	1.892 (0.758, 3.026)	0.001	1.756 (0.689, 2.823)	0.002	1.587 (0.568, 2.606)	0.002
	Group 3	3.258 (1.987, 4.529)	<0.001	3.015 (1.856, 4.174)	<0.001	2.789 (1.685, 3.893)	<0.001
	Group 4	5.689 (3.987, 7.391)	<0.001	5.215 (3.689, 6.741)	<0.001	4.892 (3.458, 6.326)	<0.001
GGT	Group 2	3.258 (1.892, 4.624)	<0.001	3.015 (1.785, 4.245)	<0.001	2.789 (1.658, 4.020)	<0.001
	Group 3	4.892 (3.258, 6.526)	<0.001	4.564 (3.089, 6.039)	<0.001	4.125 (2.858, 5.392)	<0.001
	Group 4	8.785 (6.892, 10.678)	<0.001	8.158 (6.389, 9.927)	<0.001	7.564 (5.892, 9.236)	<0.001
FLI	Group 2	0.892 (0.458, 1.326)	0.001	0.815 (0.402, 1.228)	0.001	0.748 (0.356, 1.140)	0.002
	Group 3	1.568 (0.987, 2.149)	<0.001	1.425 (0.878, 2.002)	<0.001	1.289 (0.785, 1.793)	<0.001
	Group 4	2.895 (2.158, 3.632)	<0.001	2.687 (1.985, 3.389)	<0.001	2.458 (1.802, 3.114)	<0.001

Note: Group 2: high DII (T3) and non-HFD; Group 3: low DII (T1) and HFD; Group 4: high DII (T3) and HFD. WBC: White blood cell. CRP: *C*-reactive protein. ALT: Alanine aminotransferase. AST: Aspartate aminotransferase. GGT: Gamma-glutamyl transferase. FLI: Fatty liver index. Model 1 was adjusted for demographic characteristics (age, sex, race, and educational). Model 2 was additionally adjusted for lifestyle factors (smoking, alcohol use, and physical activity). Model 3 was further adjusted for BMI, diabetes, and hypertension status. Group 1 was used as the reference.

**Table 3:** Weighted multivariable logistic regression models for outcomes by groups.

Variables	Groups	Model 1 OR (95% CI)	*p*	Model 2 OR (95% CI)	*p*	Model 3 OR (95% CI)	*p*
Elevated liver enzymes	Group 2	1.89 (1.32, 2.70)	0.001	1.76 (1.24, 2.50)	0.002	1.62 (1.15, 2.28)	0.006
	Group 3	2.54 (1.81, 3.57)	<0.001	2.32 (1.67, 3.22)	<0.001	2.08 (1.51, 2.87)	<0.001
	Group 4	4.36 (3.12, 6.10)	<0.001	3.98 (2.86, 5.53)	<0.001	3.58 (2.54, 4.98)	<0.001
Steatosis or fibrosis	Group 2	1.73 (1.25, 2.40)	0.001	1.61 (1.16, 2.23)	0.004	1.48 (1.07, 2.05)	0.018
	Group 3	2.29 (1.65, 3.17)	<0.001	2.07 (1.49, 2.88)	<0.001	1.85 (1.34, 2.55)	<0.001
	Group 4	3.87 (2.80, 5.35)	<0.001	3.52 (2.56, 4.84)	<0.001	3.12 (2.25, 4.32)	<0.001

Note: Group 2: high DII (T3) and non-HFD; Group 3: low DII (T1) and HFD; Group 4: high DII (T3) and HFD. Model 1 was adjusted for demographic characteristics (age, sex, race, and educational). Model 2 was additionally adjusted for lifestyle factors (smoking, alcohol use, and physical activity). Model 3 was further adjusted for BMI, diabetes, and hypertension status. Group 1 was used as the reference.

**Table 4:** Results of multiplicative interaction tests between DII and high-fat diet.

Variables	Interaction Terms	Model 3 OR (95% CI)	*p*
Elevated liver enzymes	DII × HFD	1.45 (1.18, 1.78)	0.001
Steatosis or fibrosis	DII × HFD	1.38 (1.12, 1.69)	0.003

**Table 5:** Sensitivity analyses.

Variables	Outcome	Main Analysis OR (95% CI)	Sensitivity Analysis OR (95% CI)	*p*
DII from the second-day dietary recall	CRP	0.764 (0.558~0.970)	0.732 (0.521~0.943)	<0.001
	ALT	5.689 (3.987~7.391)	5.426 (3.758~7.094)	<0.001
	FLI	2.458 (1.802~3.114)	2.315 (1.689~2.941)	<0.001
Including participants with liver disease	CRP	0.764 (0.558~0.970)	0.718 (0.503~0.933)	<0.001
	ALT	5.689 (3.987~7.391)	5.132 (3.401~6.863)	<0.001
	FLI	2.458 (1.802~3.114)	2.207 (1.579~2.835)	<0.001
Energy-adjusted DII	CRP	0.764 (0.558~0.970)	0.749 (0.536~0.962)	<0.001
	ALT	5.689 (3.987~7.391)	5.517 (3.829~7.205)	<0.001
	FLI	2.458 (1.802~3.114)	2.386 (1.742~3.030)	<0.001
Composite exposure as a continuous term	CRP		0.012 (0.008~0.016)	<0.001
	ALT		0.185 (0.132~0.238)	<0.001
	FLI		0.049 (0.035~0.063)	<0.001

Note: CRP, *C*-reactive protein; ALT, Alanine aminotransferase; FLI, Fatty liver index.

**Table 6:** Total weight gain, food intake and TFER.

Variables	CON	MOD	LHP	MHP	HHP	*p*
Total Weight Gain (g)	175.66 ± 26.46 ^a^	226.29 ± 23.62 ^b^	221.98 ± 41.19 ^b^	215.12 ± 17.21 ^b^	205.46 ± 25.52 ^ab^	<0.01
Total Food Intake (g)	1360.45 ± 49.47 ^a^	1234.59 ± 57.92 ^b^	1269.83 ± 78.53 ^b^	1244.77 ± 52.37 ^b^	1223.01 ± 40.58 ^b^	<0.01
Total energy (kcal)	5142.50 ± 187.00 ^a^	5889.01 ± 276.27 ^b^	6057.10 ± 374.58 ^b^	5937.54 ± 249.78 ^b^	5833.75 ± 193.56 ^b^	<0.01
TFER (%)	7.88 ± 1.06 ^a^	5.50 ± 0.48 ^b^	5.90 ± 1.16 ^b^	5.81 ± 0.43 ^b^	6.02 ± 0.67 ^b^	<0.01

Note: TFER, total food efficiency ratio. Different letters in each row indicate statistically significant differences among groups (*p* < 0.05).

**Table 7:** Inflammation levels and barrier integrity in colon organoids.

Variables	CON	MOD	ATX	*p*
TEER (Ω*cm^2^)	220.71 ± 5.39 ^a^	−11.94 ± 2.52 ^b^	−9.71 ± 0.87 ^b^	<0.001
Yellow P (10^−6^ cm/s)	1.53 ± 0.14 ^a^	47.45 ± 4.60 ^b^	33.73 ± 7.22 ^c^	<0.001
ZO-1	1.07 ± 0.51	1.58 ± 0.21	1.65 ± 0.29	0.18
Ocln	1.05 ± 0.39	1.32 ± 0.33	1.30 ± 0.29	0.58
TNF-α	1.03 ± 0.33 ^a^	6.17 ± 3.01 ^b^	4.29 ± 1.12 ^a,b^	0.04
IL-1β	1.02 ± 0.24 ^a^	9.88 ± 2.15 ^b^	18.52 ± 4.10 ^c^	<0.001
IL-6	0.95 ± 0.23	3.00 ± 2.32	3.47 ± 0.65	0.14

Note: TEER, transepithelial electrical resistance; ZO-1, zonula occludens-1; Ocln, occludin; TNF-α, tumor necrosis factor alpha; IL-1β, interleukin 1 beta; IL-6, interleukin 6. Different letters in each row indicate statistically significant differences among groups (*p* < 0.05).

## Data Availability

The NHANES data were publicly available. Animal and organoid experimental data are available by contacting the corresponding author upon reasonable request.

## Supplementary Materials

The following supporting information can be downloaded at: https://www.mdpi.com/article/10.3390/nu18071048/s1. Section S1: Supplementary Methods. Table S1. Serum Biochemical Profiles in Rats. Table S2. Lipid levels and oxidative stress status in liver tissue. Table S3. Colonic inflammation levels and barrier integrity.

## Abbreviations

The following abbreviations are used in this manuscript:

ALT	Alanine aminotransferase
AST	Aspartate aminotransferase
ATX	Astaxanthin
BMI	Body mass index
BW	Body weight
CON	Control group
CRP	*C*-reactive protein
DII	Dietary inflammatory index
DSS	Dextran sulfate sodium
FLI	Fatty liver index
GGT	Gamma-glutamyl transferase
GSH	Reduced glutathione
HDL-C	High-density lipoprotein cholesterol
HE	Hematoxylin-eosin
HFD	High-fat diet
HHP	High-dose HP powder group
HP	*Haematococcus pluvialis*
IL-10	Interleukin-10
IL-1β	Interleukin-1β
IL-6	Interleukin-6
LDL-C	Low-density lipoprotein cholesterol
LHP	Low-dose HP powder group
LPS	Lipopolysaccharide
MDA	Malondialdehyde
MHP	Medium-dose HP powder group
MOD	Model group
NHANES	National Health and Nutrition Examination Survey
Ocln	Occludin
PA	Palmitic acid
PBMCs	Peripheral blood mononuclear cells
SOD	Superoxide dismutase
TC	Total cholesterol
TEER	Transepithelial electrical resistance
TG	Triglycerides
TNF-α	Tumor necrosis factor-α
WBC	White blood cell
ZO-1	Zonula occludens-1

## References

[1] Amini-Salehi E., Letafatkar N., Norouzi N., Joukar F., Habibi A., Javid M., Sattari N., Khorasani M., Farahmand A., Tavakoli S. (2024). Global Prevalence of Nonalcoholic Fatty Liver Disease: An Updated Review Meta-Analysis comprising a Population of 78 million from 38 Countries. Arch. Med. Res..

[2] Hracs L., Windsor J.W., Gorospe J., Cummings M., Coward S., Buie M.J., Quan J., Goddard Q., Caplan L., Markovinović A. (2025). Global evolution of inflammatory bowel disease across epidemiologic stages. Nature.

[3] Eilam Y., Khattib H., Pintel N., Avni D. (2023). Microalgae-Sustainable Source for Alternative Proteins and Functional Ingredients Promoting Gut and Liver Health. Glob. Chall..

[4] Xu J., Xie L., Fan R., Shi X., Xu W., Dong K., Ma D., Yan Y., Zhang S., Sun N. (2025). The role of dietary inflammatory index in metabolic diseases: The associations, mechanisms, and treatments. Eur. J. Clin. Nutr..

[5] Shivappa N., Steck S.E., Hurley T.G., Hussey J.R., Hébert J.R. (2014). Designing and developing a literature-derived, population-based dietary inflammatory index. Public Health Nutr..

[6] Hua R., Liang G., Yang F. (2024). Meta-analysis of the association between dietary inflammation index and C-reactive protein level. Medicine.

[7] Xiong L., Diwakarla S., Chatzis R., Artaiz O., Macowan M., Zhang S., Garnham A., Morgan P.K., Mellett N.A., Meikle P.J. (2025). Acute exposure to high-fat diet impairs ILC3 functions and gut homeostasis. Immunity.

[8] Malesza I.J., Malesza M., Walkowiak J., Mussin N., Walkowiak D., Aringazina R., Bartkowiak-Wieczorek J., Mądry E. (2021). High-Fat, Western-Style Diet, Systemic Inflammation, and Gut Microbiota: A Narrative Review. Cells.

[9] Kang G.G., Trevaskis N.L., Murphy A.J., Febbraio M.A. (2023). Diet-induced gut dysbiosis and inflammation: Key drivers of obesity-driven NASH. Iscience.

[10] EASL, EASD, EASO (2024). EASL-EASD-EASO Clinical Practice Guidelines on the Management of Metabolic Dysfunction-Associated Steatotic Liver Disease (MASLD). Obesity Facts.

[11] Yang X., Guo H., Zou M. (2026). Inflammatory bowel diseases: Pathological mechanisms and therapeutic perspectives. Mol. Biomed..

[12] Du Z., Sun Y., Zhu X., Liang M., Shi D., Zhang C., Ji C., Cui H., Xue J., Li R. (2025). Astaxanthin Alleviates Lead-Induced Toxicity by Restoring Hepatic and Gut-Liver Axis Homeostasis Through Multidimensional Metabolic and Antioxidative Pathways. Food Sci. Nutr..

[13] Sun M., Xu L., Chen Q., Lu Z., Rao H., Zhao X., Chen O., Wang Y. (2026). Multistage oral astaxanthin targeted delivery system: Increasing serotonin levels with a dual therapeutic effect for alleviating inflammatory bowel disease and psychiatric disorders. Colloids Surf. B.

[14] Yang S., Lu X., Wang J., Liu Y., Nie M., Liu J., Sun H. (2025). Astaxanthin from Haematococcus pluvialis and Chromochloris zofingiensis: Biosynthetic Pathways, Engineering Strategies, and Industrial Prospects. Mar. Drugs.

[15] Zhang X., Liu R., Chen Y., Wang H., Su W., Song Y., Tan M. (2025). Dual-Targeted Nanoparticles Hitchhiking on Lactobacillus rhamnosus Bacterial Ghosts to Alleviate Nonalcoholic Steatohepatitis. ACS Nano.

[16] Wallerer S., Papakonstantinou T., Morze J., Stadelmaier J., Kiesswetter E., Gorenflo L., Barbaresko J., Szczerba E., Neuenschwander M., Bell W. (2024). Association between substituting macronutrients and all-cause mortality: A network meta-analysis of prospective observational studies. Eclinicalmedicine.

[17] Yu H., Jiang H., Li M., Yang B., Smayi A., Chen J., Wu B., Yang Y. (2023). Lowering the threshold of alanine aminotransferase for enhanced identification of significant hepatic injury in chronic hepatitis B patients. World J. Gastroenterol..

[18] Bedogni G., Bellentani S., Miglioli L., Masutti F., Passalacqua M., Castiglione A., Tiribelli C. (2006). The Fatty Liver Index: A simple and accurate predictor of hepatic steatosis in the general population. BMC Gastroenterol..

[19] Angulo P., Hui J.M., Marchesini G., Bugianesi E., George J., Farrell G.C., Enders F., Saksena S., Burt A.D., Bida J.P. (2007). The NAFLD fibrosis score: A noninvasive system that identifies liver fibrosis in patients with NAFLD. Hepatology.

[20] National Health Commission of China Announcement on the Approval of New Resource Foods such as *Haematococcus pluvialis* (No. 17, 2010). https://www.nhc.gov.cn/sps/c100088/201011/65417d81ac5c423eb33ad2021aa198ad.shtml.

[21] State Administration for Market Regulation, National Health Commission, National Administration of Traditional Chinese Medicine (2023). Technical Guidelines for Functional Testing and Evaluation of Health Foods. https://www.samr.gov.cn/zw/zfxxgk/fdzdgknr/tssps/art/2023/art_491d5c9de75e425c8cd0203027af1d93.html.

[22] Zhou Y., Feng Y., Yang L., Zheng P., Hang L., Jiang F., Yuan J., Zhu L. (2022). High-fat diet combined with dextran sulfate sodium failed to induce a more serious NASH phenotype than high-fat diet alone. Front. Pharmacol..

[23] Tian T., Zhang J., Xie W., Ni Y., Fang X., Liu M., Peng X., Wang J., Dai Y., Zhou Y. (2022). Dietary Quality and Relationships with Metabolic Dysfunction-Associated Fatty Liver Disease (MAFLD) among United States Adults, Results from NHANES 2017-2018. Nutrients.

[24] Zhang Z., Yuan Y., Hu L., Tang J., Meng Z., Dai L., Gao Y., Ma S., Wang X., Yuan Y. (2023). ANGPTL8 accelerates liver fibrosis mediated by HFD-induced inflammatory activity via LILRB2/ERK signaling pathways. J. Adv. Res..

[25] Sharma S., Le Guillou D., Chen J.Y. (2023). Cellular stress in the pathogenesis of nonalcoholic steatohepatitis and liver fibrosis. Nat. Rev. Gastroenterol. Hepatol..

[26] Ma Y., Lee G., Heo S., Roh Y. (2021). Oxidative Stress Is a Key Modulator in the Development of Nonalcoholic Fatty Liver Disease. Antioxidants.

[27] Heidari M., Chaboksafar M., Alizadeh M., Sohrabi B., Kheirouri S. (2023). Effects of Astaxanthin supplementation on selected metabolic parameters, anthropometric indices, Sirtuin1 and TNF-α levels in patients with coronary artery disease: A randomized, double-blind, placebo-controlled clinical trial. Front. Nutr..

[28] Wang M., Xu W., Yu J., Liu Y., Ma H., Ji C., Zhang C., Xue J., Li R., Cui H. (2022). Astaxanthin From Haematococcus pluvialis Prevents High-Fat Diet-Induced Hepatic Steatosis and Oxidative Stress in Mice by Gut-Liver Axis Modulating Properties. Front. Nutr..

[29] Fornari Laurindo L., Dogani Rodrigues V., Penna Carneiro D., Sérgio Marangão Filho L., Pereira E.D.S.B., José Tofano R., Chagas E.F.B., Dos Santos Haber J.F., Cristina Castilho Caracio F., Moreira L.Z. (2025). Assessing the Effects of Moderate to High Dosage of Astaxanthin Supplementation on Lipid Profile Parameters-A Systematic Review and Meta-Analysis of Randomized Controlled Studies. Pharmaceuticals.

[30] Huan Y., Zhang W., Xue L., Tang Q., Wang Y., Xu J., Xue C. (2025). Astaxanthin and astaxanthin-responsive microbial consortia synergistically ameliorate colitis in Mice: A new strategy for precision nutrition. Food Biosci..

[31] Arumugam P., Saha K., Nighot P. (2025). Intestinal Epithelial Tight Junction Barrier Regulation by Novel Pathways. Inflamm. Bowel Dis..

[32] Chen S., Wang J., Feng J., Xuan R. (2023). Research progress of Astaxanthin nano-based drug delivery system: Applications, prospects and challenges?. Front. Pharmacol..

[33] Nair A.B., Jacob S. (2016). A simple practice guide for dose conversion between animals and human. J. Basic Clin. Pharm..

[34] Lin Y., Lin J., Wang D., Chen C., Chiou M. (2017). Safety assessment of astaxanthin derived from engineered Escherichia coli K-12 using a 13-week repeated dose oral toxicity study and a prenatal developmental toxicity study in rats. Regul. Toxicol. Pharmacol..

[35] Shatoor A.S., Al Humayed S., Almohiy H.M. (2022). Astaxanthin attenuates hepatic steatosis in high-fat diet-fed rats by suppressing microRNA-21 via transactivation of nuclear factor erythroid 2-related factor 2. J. Physiol. Biochem..

[36] Yilmaz A.S., Badak B., Erkasap N., Ozkurt M., Colak E. (2023). The Effect of Antioxidant Astaxanthin on Intestinal Ischemia Reperfusion Damage in Rats. J. Investig. Surg..

[37] Wei M., Wu X., Lin J., Huang J., Cheng J., Huang W., Xu G., Yi L. (2026). Astaxanthin alleviates DSS-induced ulcerative colitis in mice associated with Nrf2-mediated ferroptosis independently of gut microbiota modulation. J. Nutr. Biochem..

[38] Zhang W., Zhang X., Lv X., Qu A., Liang W., Wang L., Zhao P., Wu Z. (2024). Oral Delivery of Astaxanthin via Carboxymethyl Chitosan-Modified Nanoparticles for Ulcerative Colitis Treatment. Molecules.

