# Supplementary material for: Synergistic Effects of a Pro-Inflammatory–High-Fat Composite Dietary Pattern on Gut–Liver Injury and the Therapeutic Potential of Haematococcus pluvialis-Derived Astaxanthin
Source: Nutrients. 2026 Mar 25;18(7):1048. doi: 10.3390/nu18071048 (PMC13074625; doi:10.3390/nu18071048)
Supplement: Supplementary file 1 [file nutrients-18-01048-s001.zip › nutrients-4207286-supplementary.pdf]

Synergistic Effects of a Pro-Inflammatory–High-Fat Composite Dietary Pattern on Gut–Liver Injury and the Therapeutic Potential of *Haematococcus pluvialis*-Derived Astaxanthin

Jing Feng, Chao Han, Jinpeng Zhao, Zhuo Yang, Chen Chen, Rongzi Li, Chaoqun Sun, Liyuan Wang, Junsheng Huo, Shi Shen and Qin Zhuo *

Key Laboratory of Public Nutrition and Health, National Institute for Nutrition and Health, Chinese Center for Disease Control and Prevention, Beijing 100050, China; fengjing0921@163.com (J.F.); hanchao@ninh.chinacdc.cn (C.H.); zhaojp@ninh.chinacdc.cn (J.Z.); yangzhuo@ninh.chinacdc.cn (Z.Y.); chenchen@ninh.chinacdc.cn (C.C.); lirz@ninh.chinacdc.cn (R.L.); suncq@ninh.chinacdc.cn (C.S.); wangly@ninh.chinacdc.cn (L.W.); huojs@ninh.chinacdc.cn (J.H.); shenshi@ninh.chinacdc.cn (S.S.)

* Correspondence: zhuoqin@ninh.chinacdc.cn; Tel.: +86(10)-66237240

List of Supplementary Materials

1.Supplementary Methods 2

1.1. Culture and maintenance of colonic organoids 2

1.2. Construction of the colonic barrier 2

1.3. Establishment and treatment of the intestinal organoid－immune cell barrier model 2

1.4. Measurement of TEER 2

1.5. Measurement of Lucifer Yellow Papp 3

1.6. Analysis of tight junction proteins and inflammatory factors in intestinal organoids 3

2.Supplementary Tables 3

Table S1. Serum Biochemical Profiles in Rats 3

Table S2. Lipid levels and oxidative stress status in liver tissue 4

Table S3. Colonic inflammation levels and barrier integrity 4

1. Supplementary Methods

1.1. Culture and maintenance of colonic organoids

Cryopreserved colonic organoids were retrieved from liquid nitrogen and thawed in a 37°C water bath. The organoids were then transferred into a centrifuge tube containing coating solution, gently mixed, and centrifuged to collect the organoid pellet. The pellet was resuspended in matrix gel and seeded as droplets into a 24-well plate. After gel solidification in an incubator, 500 μL of intestinal organoid expansion medium was added to each well to initiate organoid culture. During the first 2 days of culture, an anti-apoptotic factor was supplemented into the medium. The culture medium was replaced every 3~4 days, and organoids were passaged every 4~7 days according to their growth status.

1.2. Construction of the colonic barrier

Prior to organoid seeding, the IBAC M1 organ chip was treated with 1× modification solution diluted in DPBS and incubated for more than 2 h to coat the inner surface of the chip with proteins. After coating, the chip was rinsed with DMEM/F12 medium and prepared for subsequent use.

To prepare cells for seeding, organoid washing solution was added to the wells, and the organoid–matrix gel mixture was gently dissociated by pipetting and collected into a centrifuge tube. Following centrifugation, the cell pellet was resuspended in organoid digestion solution, and the organoids were mechanically dissociated by repeated pipetting. The digestion was stopped by adding organoid washing solution, followed by centrifugation to recollect the cells. The resulting colonic organoid pellet was resuspended in culture medium containing an anti-apoptotic factor and seeded onto the porous membrane surface of the apical channel layer (the lower side of the M1 chip).

After cell attachment and spreading, intestinal organoid expansion medium was added to initiate colonic barrier culture. Once the colonic epithelial cells had proliferated and formed a confluent barrier, the medium was replaced with intestinal organoid differentiation medium. During culture, the IBAC Rocker system was used to provide dynamic culture conditions, and fluid shear stress was applied to promote structural and functional maturation of the colonic barrier. After 8 days of culture, the colonic barrier model was used for subsequent co-culture experiments and intervention studies.

1.3. Establishment and treatment of the intestinal organoid – *immune cell barrier model*

Following establishment of the colonic barrier, peripheral blood mononuclear cells (PBMCs) were thawed on the same day and seeded onto the basolateral side of the colonic barrier (the upper side of the M1 chip) at a density of 5 × 10^4 cells/well to establish a colon–PBMC co-culture system. In this system, the PBMC compartment was maintained in PBMC culture medium, which was replaced every 2 days, whereas the colonic compartment was maintained in intestinal organoid differentiation medium, which was refreshed daily.

After establishment of the colon–PBMC co-culture system, barrier injury was induced with palmitic acid (PA) and lipopolysaccharide (LPS), while ATX was administered to the colonic side for 5 days. At the end of treatment, PBMCs and supernatants from the PBMC side were removed. Transepithelial electrical resistance (TEER) and the apparent permeability coefficient (Papp) of Lucifer Yellow were then measured to evaluate barrier integrity and permeability. In addition, colonic organoid lysates were collected for analysis of the transcriptional levels of tight junction proteins and inflammatory factors.

1.4. Measurement of TEER

The culture medium was removed from all wells of the M1 chip, and each unit was washed with Hank's balanced salt solution (HBSS). Subsequently, 100 μL and 200 μL HBSS

were added to the basolateral and apical sides of the chip, respectively. The transepithelial electrical resistance of each well was measured using a resistance meter. TEER values were calculated according to Equation 1.

$$\text{TEER } (\Omega{*}\text{cm}^2) = (R_{\text{Sample}}\text{-}R_{\text{Black}}) \times A \qquad \text{Equation 1}$$

R_{Sample}: the total resistance of the sample well (Ω); R_{Blank}: the resistance of the blank well (Ω); A: the membrane surface area (cm^2).

1.5. Measurement of Lucifer Yellow Papp

The culture medium in each well was discarded, and the wells were washed with HBSS. Then, 110 μL HBSS and 220 μL Lucifer Yellow working solution were added to the basolateral and apical sides of the chip, respectively. The chip was incubated in an incubator for 3 h. After incubation, 100 μL of supernatant from the basolateral side of each well was collected and transferred into a black 96-well plate for fluorescence measurement.

A standard curve was prepared by serial 1:2 dilution of the Lucifer Yellow working solution with HBSS to generate 16 concentration points, and two wells containing HBSS alone were used as solvent blank controls. Fluorescence intensity was measured using a microplate reader at an excitation wavelength of 428 nm and an emission wavelength of 536 nm, with the gain set to 100. The concentration of Lucifer Yellow in each sample was calculated from the standard curve, and the apparent permeability coefficient (Papp) was calculated according to Equation 2.

$$P_{app}\ (\text{cm/s}) = \frac{1}{A \times C_0} \times \frac{C_S \times V}{t} \qquad \text{Equation 2}$$

A: the membrane surface area (cm^2); C_0: the concentration of the Lucifer Yellow working solution (μg/mL); C_S: the Lucifer Yellow concentration on the basolateral side (μg/mL); V: the sampling volume (mL); t: the incubation time (s).

1.6. Analysis of tight junction proteins and inflammatory factors in intestinal organoids

Total RNA was extracted from colonic organoids using a total RNA extraction kit for micro-samples according to the manufacturer's instructions. Briefly, colonic organoids were lysed using the cell lysis buffer provided in the kit, followed by RNA extraction and washing to obtain total RNA. The extracted RNA was reverse-transcribed into cDNA using HiScript III RT SuperMix. Quantitative real-time PCR (RT-qPCR) was then performed using ChamQ Universal SYBR qPCR Master Mix together with gene-specific primers and sample cDNA.

The amplification protocol consisted of an initial pre-denaturation step at 95°C for 30 s, followed by 40 cycles of denaturation at 95°C for 10 s and annealing/extension at 60°C for 30 s. Primer sequences for the target genes are listed in the array below.

Primer sequences

	Forward Primer	Reversed Primer
IL-1β	AGCTACGAATCTCCGACCAC	CGTTATCCCATGTGTCGAAGAA
IL-6	ACTCACCTCTTCAGAACGAATTG	CCATCTTTGGAAGGTTCAGGTTG
TNF-α	GAGGCCAAGCCCTGGTATG	CGGGCCGATTGATCTCAGC
ZO-1	AGGAGAGGTGTTCCGTGTTGTG	GCTCTGTTCTTATTAGGGATGATGC
Ocln	CACCCCCATCTGACTATGTGGAAAG	ACCGCTGCTGTAACGAGGCTG

2. Supplementary Tables

Table S1. Serum Biochemical Profiles in Rats

Variables	CON	MOD	LHP	MHP	HHP	*p*
TG	0.59±0.19	0.74±0.20	0.75±0.23	0.73±0.21	0.77±0.35	0.50

(mmol/L)						
TC (mmol/L)	1.97±0.25 [a]	2.39±0.27 [b]	2.22±0.30 [ab]	2.14±0.21 [ab]	2.14±0.24 [ab]	0.07
LDL-C (mmol/L)	0.39±0.05 [a]	0.56±0.12 [b]	0.39±0.03 [a]	0.44±0.05 [b]	0.41±0.04 [a]	< 0.01
HDL-C (mmol/L)	1.38±0.16 [a]	0.89±0.16 [b]	1.54±0.23 [ac]	1.65±0.16 [c]	1.60±0.15 [ac]	< 0.01
ALT (U/L)	28.8±3.8 [a]	55.7±21.8 [b]	37.0±6.8 [ab]	39.1±6.0 [b]	35.7±7.3 [ab]	< 0.01
AST (U/L)	146.5±29.2 [a]	201.4±47.5 [b]	148.1±29.6 [a]	146.5±21.9 [a]	145.4±21.8 [a]	< 0.01

Note: TG, triglycerides; TC, total cholesterol; LDL-C, low-density lipoprotein cholesterol; HDL-C, high-density lipoprotein cholesterol; AST, aspartate aminotransferase; ALT, alanine transaminase. Different letters in each row indicate statistically significant differences among groups ($p < 0.05$).

Table S2. Lipid levels and oxidative stress status in liver tissue

**Variables**	**CON**	**MOD**	**LHP**	**MHP**	**HHP**	***p***
Liver Weight (g)	11.28±0.85 [a]	17.70±2.18 [b]	12.38±1.15 [a]	12.26±0.63 [a]	12.03±0.83 [a]	< 0.01
Liver Index	2.20±0.08 [a]	3.14±0.40 [b]	2.21±0.10 [a]	2.20±0.09 [a]	2.21±0.16 [a]	< 0.01
Liver Score	1.0±1.4 [a]	5.8±1.6 [b]	0.9±1.2 [a]	1.0±1.3 [a]	1.2±1.3 [a]	< 0.01
Hepatic TG (mmol/g prot)	65.76±16.43 [a]	208.64±26.86 [b]	88.91±30.25 [a]	93.81±33.48 [a]	93.43±31.00 [a]	< 0.01
Hepatic TC (mmol/g prot)	23.95±2.66 [a]	97.80±11.63 [b]	29.28±4.40 [ac]	31.71±6.48 [c]	29.98±5.94 [c]	< 0.01
MDA (nmol/mg prot)	0.18±0.04 [a]	0.31±0.08 [b]	0.20±0.03 [a]	0.21±0.03 [a]	0.21±0.04 [a]	< 0.01
Protein Carbonyl (nmol/mg prot)	1.08±0.29 [a]	1.90±0.25 [b]	0.99±0.21 [a]	1.24±0.33 [a]	0.99±0.44 [a]	< 0.01
SOD (U/mg prot)	10.35±0.73 [a]	6.16±0.55 [b]	9.48±0.48 [c]	9.92±0.72 [ac]	9.83±0.63 [ac]	< 0.01
GSH (μg/mg prot)	8.63±1.02 [a]	5.65±1.09 [b]	6.93±1.13 [bc]	7.37±0.92 [ac]	7.35±1.06 [c]	< 0.01

Note: TG, triglycerides; TC, total cholesterol; MDA, malondialdehyde; SOD, superoxide dismutase; GSH, reduced glutathione. Different letters in each row indicate statistically significant differences among groups ($p < 0.05$).

Table S3. Colonic inflammation levels and barrier integrity

**Variables**	**CON**	**MOD**	**LHP**	**MHP**	**HHP**	***p***
Colon Weight (g)	1.44±0.20	1.36±0.10	1.33±0.13	1.44±0.21	1.36±0.16	0.52
Colon Index	0.28±0.04	0.24±0.02	0.24±0.03	0.26±0.04	0.25±0.03	0.04
Colon Score	0.0±0.0 [a]	5.6±3.7 [b]	3.3±3.2 [b]	1.7±1.9 [a]	1.1±1.8 [a]	0.04

ZO-1 (pg/mg prot)	71.79±12.90 [a]	42.63±16.44 [b]	55.46±10.09 [ab]	56.12±12.03 [ab]	60.46±8.95 [ab]	< 0.01
Ocln (pg/mg prot)	138.24±29.61 [a]	96.77±33.08 [b]	123.41±16.08 [ab]	116.75±11.38 [ab]	123.68±16.89 [ab]	0.02
TNF-α (pg/mg prot)	33.94±14.07 [a]	66.65±16.79 [b]	36.41±14.39 [a]	32.03±11.19 [a]	28.18±13.21 [a]	< 0.01
IL-1β (pg/mg prot)	4.32±0.69 [a]	7.03±0.97 [b]	5.20±0.71 [a]	5.23±0.83 [a]	4.82±1.08 [a]	< 0.01
IL-6 (pg/mg prot)	32.18±4.85 [a]	56.29±9.42 [b]	39.34±5.39 [a]	38.36±5.86 [a]	35.57±8.02 [a]	< 0.01
IL-10 (pg/mg prot)	15.97±2.81 [a]	7.96±4.38 [b]	10.69±2.44 [b]	12.69±2.89 [ab]	13.25±2.38 [a]	< 0.01

Note: ZO-1, zonula occludens-1; Ocln, occludin; TNF-α, tumor necrosis factor alpha; IL-1β, interleukin 1 beta; IL-6, interleukin 6; IL-10, interleukin 10. Different letters in each row indicate statistically significant differences among groups ($p < 0.05$).
